# Direct production of XY^*DMY*−^ sex reversal female medaka (*Oryzias latipes*) by embryo microinjection of TALENs

**DOI:** 10.1038/srep14057

**Published:** 2015-09-14

**Authors:** Daji Luo, Yun Liu, Ji Chen, Xiaoqin Xia, Mengxi Cao, Bin Cheng, Xuejuan Wang, Wuming Gong, Chao Qiu, Yunsheng Zhang, Christopher Hon Ki Cheng, Zuoyan Zhu, Wei Hu

**Affiliations:** 1 Department of Genetics, School of Basic Medical Sciences, Wuhan University, Wuhan, Hubei, P. R. China, 430071; 2 State Key Laboratory of Freshwater Ecology and Biotechnology, Institutes of Hydrobiology, Chinese Academy of Sciences, Wuhan, Hubei, P. R. China, 430072; 3 School of Biomedical Sciences, The Chinese University of Hong Kong, Shatin, Hong Kong, P. R. China; 4 Lillehei Heart Institute, University of Minnesota, Minneapolis, USA

## Abstract

Medaka is an ideal model for sex determination and sex reversal, such as XY phenotypically female patients in humans. Here, we assembled improved TALENs targeting the *DMY* gene and generated XY^*DMY*−^ mutants to investigate gonadal dysgenesis in medaka. *DMY*-TALENs resulted in indel mutations at the targeted loci (46.8%). *DMY-nanos3UTR*-TALENs induced mutations were passed through the germline to F1 generation with efficiencies of up to 91.7%. XY^*DMY*−^ mutants developed into females, laid eggs, and stably passed the Y^*DMY*−^ chromosome to next generation. RNA-seq generated 157 million raw reads from WT male (WT_M_TE), WT female (WT_F_OV) and XY^*DMY*−^ female medaka (TA_F_OV) gonad libraries. Differential expression analysis identified 144 up- and 293 down-regulated genes in TA_F_OV compared with WT_F_OV, 387 up- and 338 down-regulated genes in TA_F_OV compared with WT_M_TE. According to genes annotation and functional prediction, such as *Wnt1* and *PRCK*, it revealed that incomplete ovarian function and reduced fertility of XY^*DMY*−^ mutant is closely related to the wnt signaling pathway. Our results provided the transcriptional profiles of XY^*DMY*−^ mutants, revealed the mechanism between sex reversal and *DMY* in medaka, and suggested that XY^*DMY*−^ medaka was a novel mutant that is useful for investigating gonadal dysgenesis in phenotypic female patients with the 46, XY karyotype.

Over two decades have passed since the male determining gene, Sry, was identified^1^. The SRY gene plays a pivotal role in sex determination: point mutations or deletions of the SRY gene are found in approximately 15% of XY females in humans^2^,^3^. XY female syndrome, phenotypic female patients with the XY karyotype, has been studied clinically, cytogenetically, hormonally, endoscopically and histologically^4^,^5^,^6^. However, the transcriptional and post-transcriptional regulation mechanisms of SRY-induced XY female syndrome remain largely unknown. A lack of animal models has meant that it is difficult to perform in-depth studies of the pathological changes and to determine the mechanisms underlying the XY female syndrome.

Sex-determining systems in fish are very diverse, which could be determined by heredity, environment, or both. Meanwhile, the pathway of sex determination can be manipulated by administering exogenous sex steroids^7^,^8^,^9^,^10^,^11^. In non-mammalian species, which also have a XX-XY sex-determination system, SRY is not present at all. Until *DMY* gene of medaka (*Oryzias latipes*) was identified in the teleost fish^12^,^13^, it is believed that the XX-XY sex-determination system was conserved in a wide range of animals, including *C. elegans*, *Drosophila*, fish, and mammals. Although much is known about the master male sex-determining (SD) gene in medaka^12^,^14^,^15^,^16^, the precise mechanisms involved in primary sex determination and sex differentiation remain undefined. It is difficult to perform specific gene targeting in medaka, because of the lack of methodologies for homologous recombination and embryonic stem cell derivation, which has impeded its use in male heterogametic (XX-XY) sex determination system studies.

Modifications of genomes have laid the foundation of functional studies in modern biology and have led to significant discoveries^17^. Recently, Zinc finger nucleases (ZFNs)^18^,^19^,^20^, transcription activator-like effector nucleases (TALENs)^21^,^22^,^23^,^24^ and clusters of regularly interspaced short palindromic repeats (CRISPR)^25^,^26^,^27^ have been shown to edit genomic DNA in a variety of cell types and different model organisms at stable efficiency and specificity. In our previous studies, we modified the Golden Gate method to disrupt the gene and edit the genome in zebrafish^28^,^29^ and medaka^30^. Here, we directly produced TALEN-induced XY^*DMY*−^ females in medaka using TALENs. Phenotype of XY^*DMY*−^ mutant is very similar to human XY female syndrome^4^,^5^,^6^ and SRY KO mouse^3^,^31^, especially as several individuals in the population were fertile.

The specificity of targeted sites and the off-target phenomenon are core problems in gene knockout and gene editing research. To test for potential nonspecific mutations induced by TALENs, we also developed a simple and reliable off-target prediction program for confirming TALEN-induced mutation. The TALEN-induced XY^*DMY*−^ medaka developed into females and laid eggs. Although the sex-determining function of the DMY protein is already recognized; however, as a transcription factor, how does it regulate the downstream factor(s) to control testis differentiation, development and germ cell maturation? To better explain *DMY*’s regulatory functions as a transcription factor, we performed RNA-seq, a recently developed approach to transcriptome profiling based on deep-sequencing^32^, and generated 157 million raw reads from WT male (WT_M_TE), WT female (WT_F_OV) and XY^*DMY*−^ female medaka (TA_F_OV) gonad libraries. These transcriptomic data will contribute to unravel the relationship and mechanism between sex reversal and the *DMY* gene. Our results suggest that the medaka XY^*DMY*−^ mutant is a novel mutant line that is useful for investigating XY to XX sex reversal and gonadal dysgenesis in phenotypically female patient with the 46, XY karyotype.

## Results

### *DMY*- and *DMY-nanos3UTR*- TALENs effectively induced *DMY* gene disruption in Medaka

**Figure 1:**
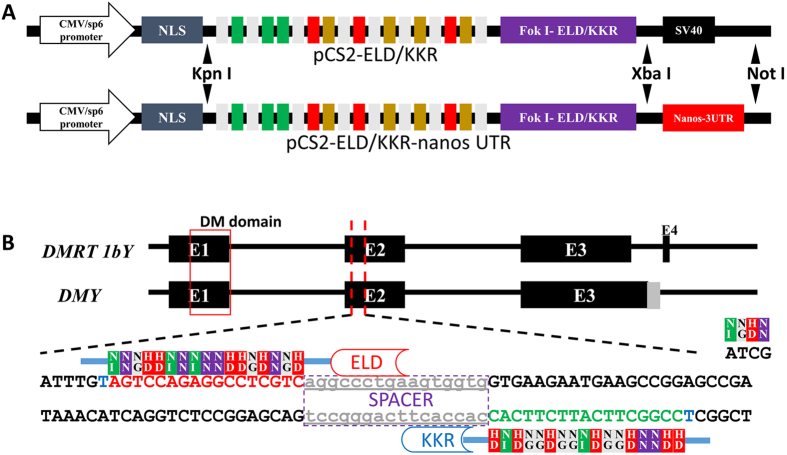
The design and assembly of *DMY-*TALEN sites. (**A**) Reconstructions of pCS2- *DMY*-TALENs -ELD/KKR-nanos UTR plasmids. (**B**) The location and sequence information of *DMY*-TALENs. Red uppercase letters indicate the DNA sequence of *DMY*-TALEN-L sites. Green uppercase letters indicate the complementary paired DNA sequence of *DMY*-TALEN-R sites. Lowercase letters indicate the spacer region of TALEN sites.

**Figure 2:**
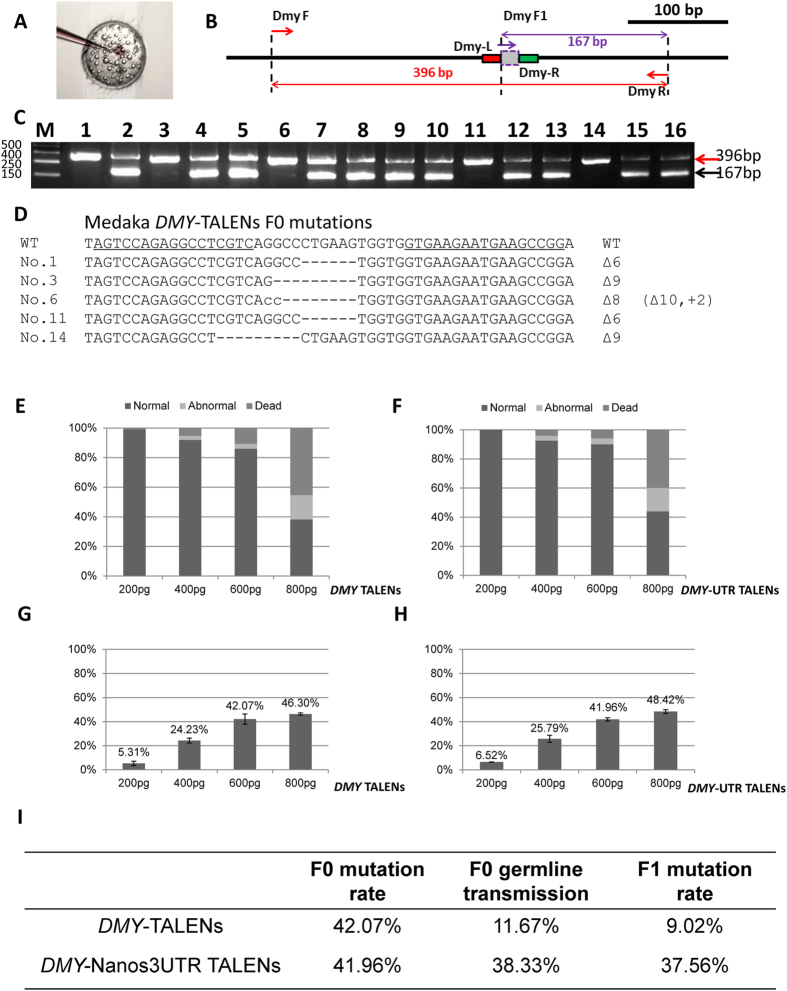
The dosage effects and efficiency evaluation of *DMY*-TALENs. (**A**) Microinjection of TALENs mRNA into medaka. (**B**) Detection of mutations in TALEN targeted medaka embryos. Primers DMY F, DMY F1 and DMY R were used to amply the *DMY* gene. Primers DMY F and DMY R bridge both EBE regions, while primers DMY F1 and DMY R link the spacer region and the downstream EBE. DMY F and DMY R generated a 396 bp PCR fragment. DMY F1 and DMY R generated a 167 bp PCR fragment. If primer DMY F and DMY R generated a PCR fragment, while primer DMY F1 and DMY R failed to do so, this suggested that the targeted gene is disrupted by the *DMY*-TALEN. (**C**) Electrophoretic detection of mutations in TALEN-injected medaka embryos. Line 1 to 16, TALENs injected embryos. 1,3,6,11,14 show mutated embryos. (**D**) Sequencing detection of mutations in TALEN-induced medaka embryos. -, deleted nucleotide; lowercase letter, added nucleotide. +, insertions; Δ, deletions. (**E**) Evaluation of embryonic toxicity of *DMY*-TALENs. (**F**) Evaluation of embryonic toxicity of *DMY*-nanos3UTR-TALENs. (**G**) The targeting efficiency statistics of *DMY*- TALENs. (**H**) The targeting efficiency statistics of *DMY*-nanos-3UTR TALENs. (**I**) Comparative analysis of mutation rate and germline transmission rate between *DMY*-TALENs and *DMY*-nanos3UTR-TALENs.

To improve the germline integration efficiency, we incorporated the zebrafish *nanos*-3′UTR into the TALEN construct (Fig. 1A). Potential TALEN target sites were scanned and designed in the exon of the *DMY*/*DMRT1bY* gene (ENSORLT000000025382 and ENSORLT000000025383) (Fig. 1B). We generated TALEN constructs using our previously published method^29^. The mixture containing a pair of TALEN mRNAs was microinjected into one-cell stage embryos of medaka (Fig. 2A and File S1). 72 hours after microinjection, ten injected embryos were randomly pooled for extracting genomic DNA. As illustrated in Fig. 2B, primers DMY F and DMY R bridge both the effector binding element (EBE) regions, while primers DMY F1 and DMY R link the spacer region and the downstream EBE. If primer DMY F and DMY R generated a 396 bp fragment, while primer DMY F1 and DMY R failed to generate the 167 bp fragment, the result suggested that the targeted gene was disrupted by the TALENs (Fig. 2C). Sequenced PCR positive clones had mutated sequences in the spacer (Fig. 2D). Both *DMY*-TALENs and *DMY*-Nanos-3UTR TALENs were effective at disrupting the targeted genes in medaka embryos. Various concentrations (200, 400, 600 and 800 pg) of the TALENs mRNA were microinjected (Fig. 2E,F, File S1 and S2). The targeting efficiency of the *DMY*-TALENs was good, with a higher TALEN-induced mutation ratio of 42.07% and lower levels of dead and deformed embryos when 600 pg mRNA was microinjected (Fig. 2G,I). Similarly, the targeting efficiency of *DMY*-nanos-3UTR TALENs was about 41.96% (Fig. 2H,I). Thus, 600 pg was determined as the appropriate concentration in medaka. These results indicated that the TALEN activity was dose-dependent, and high-dose microinjection of TALEN mRNA might cause nonspecific and toxic defects in medaka embryos (Fig. 2E–I, File S1 and S2).

Successful germline transmission is essential to establish knockout lines. To evaluate the germline transmission efficiency of the TALEN-mediated gene disruption, ten embryos for *DMY* and *DMY-*nanos3UTR from each independent cross were individually collected at 3 days post fertilization (dpf), and genomic DNA was extracted from each cross to assess mutagenesis at the TALEN-targeted site (Fig. 2C,D). 9.02% of *DMY*-TALEN-induced F1 embryos carried mutations; and 37.56% of *DMY-*nanos3UTR-TALEN-induced F1 embryos carried mutations in the *DMY* gene (Fig. 2I and File S2). The higher proportion induced by *DMY-*nanos3UTR-TALEN indicated that a majority of gametes in the F0 medaka were mutant. These results indicated that there was no significant difference in the targeting efficiency of F0 somatic mutations at the targeted loci (Fig. 2H,I); however, *DMY*-nanos3UTR-TALEN induced a higher portion of mutations in the germline than *DMY*-TALENs did.

### A novel program to identify potential off-target sites of TALENs

To test for potential nonspecific mutations induced by TALENs, we designed a program to scan the medaka genomic sequence (http://www.ensembl.org/Oryzias_latipes) to identify potential off-target sites potentially targeted by *DMY* TALENs. Potential off-target sites of *DMY*-TALENs were searched using the program and 55 candidate sites were identified (File S3). When the spacers were less than 10 bp or more than 24 bp long, the scaffold of TALENs had lower disrupting activity^33^,^34^. Five of the 55 candidates had spacers less than 10 bp, and 43 of 55 candidates had spacers of more than 24 bp, indicating that it is unlikely that the TALENs could induce mutations at these sites. We analyzed one candidate site (Chr.3: 36,197,357–36,197,403) that had 7-bp mismatches in the recognition sequences and a 12-bp spacer (File S4). PCR amplified the identified potential off-target regions using genomic DNA from TALEN-injected embryos as template; no mutations were found at these sites by DNA sequencing. This result suggested that the novel program could predict the potential off-target sites of TALENs; and that TALENs have high specificity for their target sequences.

### Mating scheme of TALENs-induced *DMY*- mutants and mutant phenotypes

**Figure 3:**
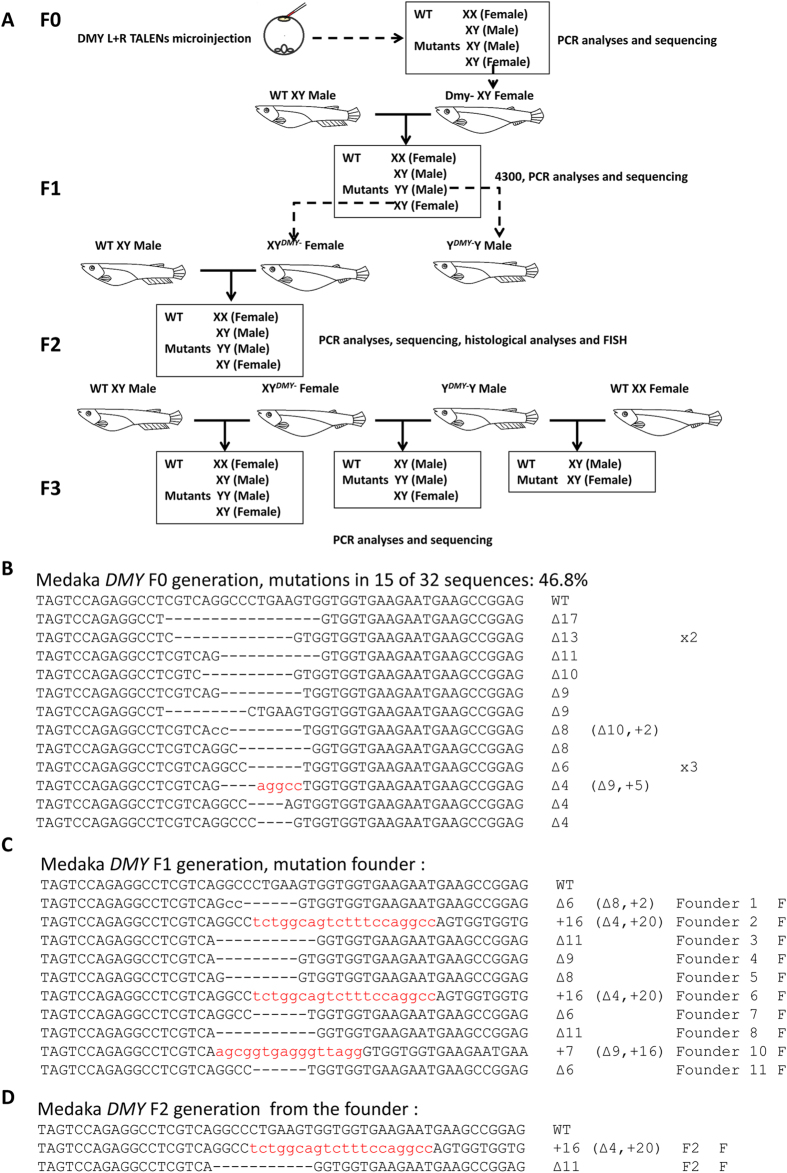
Flowchart and establishment of *DMY*-TALENs-induced mutant lines. (**A**) Flowchart of *DMY*-TALENs-induced mutant lines. (**B**) The genotypes of TALENs-induced mutations in the F0 generation. (**C**) The genotypes of TALENs induced F1 founders. (**D**) The genotypes of TALENs induced F2 mutant lines. Red lowercase letters indicate an additional nucleotide; “-” indicates a deleted nucleotide. +, insertions; Δ, deletions. F, female; M, male.

**Figure 4:**
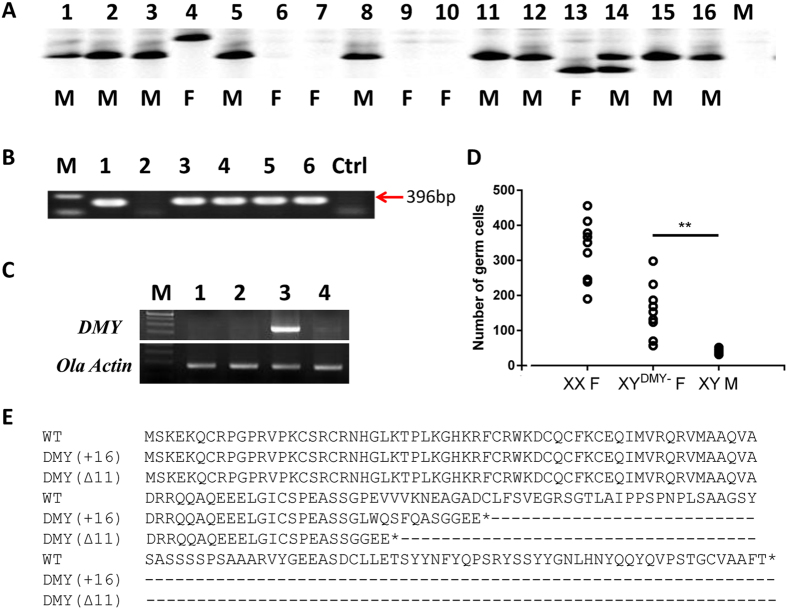
*DMY* genotyping, expression and CDS frameshift mutations of *DMY* mutants. (**A**) Genotyping of *DMY* gene fragments using Li-con 4300 system. 1 to 16, randomly selected individuals in F1 generation. F, female; M, male. (**B**) Genomic PCR confirmed the *DMY* gene. M: marker DL2000; 1: WT Male; 2: WT Female; 3: Founder 2; 4: the female mutant from the F2 generation of Founder 2; 5: Founder 3; 6: the female mutant from the F2 generation of Founder 3; Ctrl: no template control. (**C**) Reverse transcription- PCR with *DMY*-specific primers. *OLA Actin* expression was determined for calibration. M: 250 bp Marker; 1. XY^*DMY*−^ mutant (DMYΔ11); 2. XY^*DMY*−^ mutant (DMY+16); 3. WT XY male; 4. WT XX female. (**D**) Numbers of germ cells in mutants and WT medaka fry at 5DAH. XX F: WT XX females; XY^*DMY*−^ F: XY^*DMY*−^ matants; XY M: WT XY males. Open circles represent the number of germ cells in individuals (N = 9). **P < 0.001. (**E**) The CDS frameshift mutations of TALEN-induced *DMY* gene. WT: CDS sequence of DMY.

The mating scheme for the TALENs-induced *DMY*-mutant lines is shown in Fig. 3A. The F0 generation was produced by microinjecting 600 pg *DMY*-nanos3UTR-TALENs into one-cell stage embryos of medaka; the mutation rate was 46.8% (15/32) (Fig. 3B). The *DMY* gene knockout medaka could develop into females. 11-bp deletions (named DMYΔ11) and 16-bp insertions (named DMY+16) were identified and chosen to establish mutant lines (Fig. 3C,D). Notably, during the establishment of the mutant lines, two genotyping alleles of *DMY* gene fragments were identified in individual TALEN-induced F1 mutations using the Li-con 4300 system (Fig. 4A). This result indicated that Y^*DMY*−^Y male mutants were present in the testcross F1 generation. The genetic males (XY) of the *DMY* gene mutants, DMYΔ11 and DMY+16 mutant types, developed into females in the F2 generation, which was identified using genomic PCR of the *DMY* gene (Fig. 4B). To confirm un-expression and dysfunction of *DMY* gene in the XY^*DMY*−^ F2 generation, using RT-PCR, expression of *DMY* gene was not identified in the XY^*DMY*−^ female medaka (both DMYΔ11 and DMY+16) (Fig. 4C). The first morphological sex difference manifested in the gonads is reflected in the number of germ cells^14^,^35^. The number of germ cells in several DMY mutants identified from wild populations resembled that of the female^13^,^16^,^36^. To elucidate sex reversal during the development of XY^*DMY*−^ mutants, we evaluated the effect of DMY on germ cell number at 5 days after hatching (DAH) in the XY^*DMY*−^ F3 generation. The XY^*DMY*−^ mutant fry had more germ cells than that of the WT XY male at 5 DAH (Fig. 4D). This implied sex reversal of XY^*DMY*−^ mutants took place in early developmental stages, and the increased number of germ cells in the XY^*DMY*−^ mutants may be due to the disruption of *DMY* gene expression.

According to the amino acid sequence of DMY, DMYΔ11 and DMY+16 are frame-shifted mutant alleles that would produce truncated DMY protein caused by a region of altered translation (Fig. 4E). Thus, an error of the coding sequence has occurred in the mutated *DMY* gene that resulted in the loss function of the *DMY* gene. The XY^*DMY*−^ female or Y^*DMY*−^Y male (N = 10) from F2 generation that had lost the *DMY* gene were crossed with the WT to obtain the testcross F3 generation. XX female, XY male, XY^*DMY*−^ mutant female and Y^*DMY*−^Y mutant male were identified in the XY female testcross F3 generation. XY males and XY^*DMY*−^ mutant females were identified in the Y^*DMY*−^Y male testcross F3 generation. This showed that the *DMY* gene in the Y chromosome of WT medaka rescued the female phenotype of *DMY* gene disruption in mutated XY female medaka. Unfortunately, an Y^*DMY*−^Y^*DMY*−^ mutant female with a genomic homozygous *DMY* gene mutation on the Y chromosome was never found.

**Figure 5:**
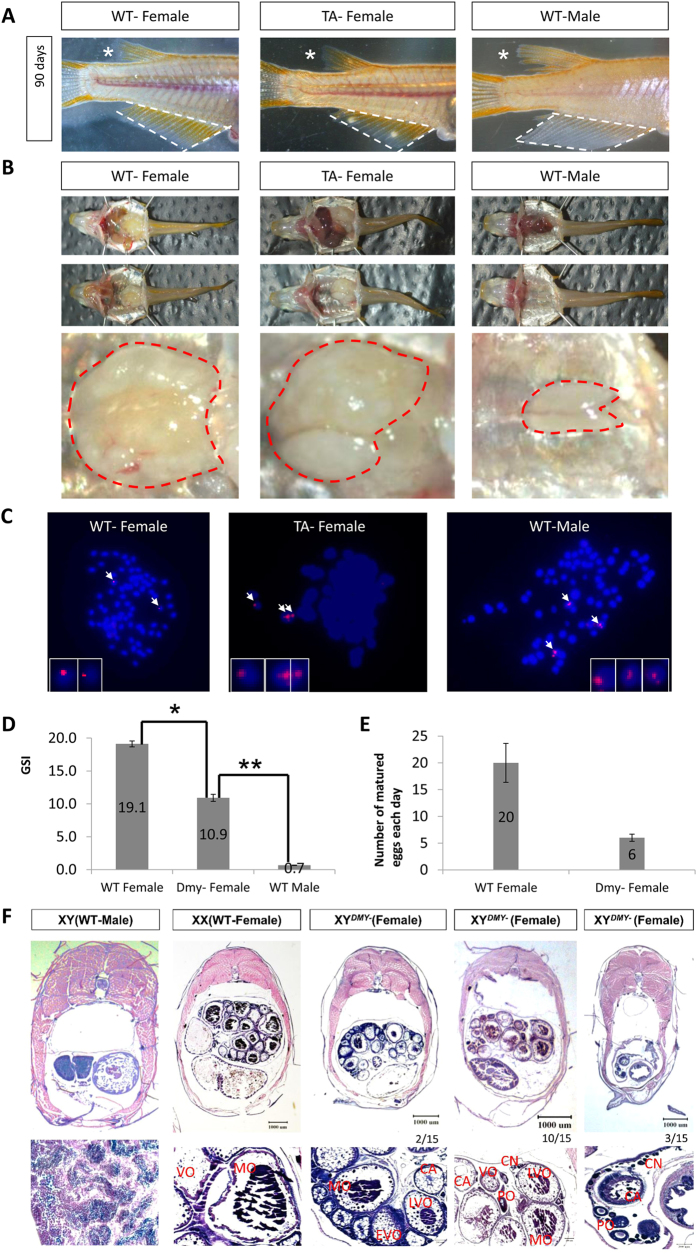
Phenotypic identification and analysis of XY^*DMY*−^ mutants. (**A**) Phenotypic diagnostics of the secondary sexual characters in XY^*DMY*−^ mutants. White dashed area shows the anal fin of medaka. *shows the dosal fin of medaka. (**B**) The gonad of WT and TA mutants. The red dashed area shows the gonad tissue of medaka. (**C**) Fluorescence *in situ* hybridization of the karyotypes of metaphases cell from WT male, WT female and XY mutant female. The pink signal is the male specific hybridization signal. (**D**) The comparative analyses of gonadosomatic index (GSI) among WT-F, WT-M and XY mutant female (TA-F). *P < 0.5; **P < 0.001. (**E**) The comparative analyses of oocytes maturation between WT-F and TA-F. (**F**) Histological analyses of gonad tissue. CN, chromatin nucleolar oocytes; PO, perinucleolar oocytes; CA, cortical alveolar oocytes; EVO, early vitellogenic oocytes; VO, vitellogenic oocytes; LVO, late vitellogenic oocytes; MO, mature oocytes.

Mature XY^*DMY*−^ mutants in the F3 generation were obtained for phenotype identification, histological analyses and fluorescence *in situ* hybridization (FISH) (Fig. 5). There are significant differences between females and males in the size and shape of dorsal and anal fins^13^,^14^, which are main part of the secondary morphological sexual characteristics in medaka. The shape of both dorsal and anal fins of XY^*DMY*−^ sex reversal female were similar to that of the WT XX female, rather than to that of the WT XY male. The size of both dorsal and anal fins of XY^*DMY*−^ female was significant smaller than those of the WT XY males (Fig. 5A). In addition, ovarian tissue was identified in XY^*DMY*−^ mutants (Fig. 5B). To confirm the Y chromosome in the TALENs-induced mutants, we analyzed the karyotypes of metaphase cells from the WT male, WT female and XY^*DMY*−^ female using FISH showing the male specific hybridization signal^12^. Compared with the two spots in females, three hybridization spots for the specific probe were identified in males (Fig. 5C). The additional FISH signal in males is on the Y chromosome. XY^*DMY*−^ mutants did not express *DMY* gene, which were different from WT XY individuals (Fig. 4C). The gonadosomatic index (GSI) and the maturation stages of oocytes are commonly used to evaluate the gonad and gonad development^37^. At 90 days after hatching, the GSI of XY^*DMY*−^ mutated female was 10.9 (N = 10); that of the WT female and WT male were 19.1 and 0.7, respectively (Fig. 5D). During the generation of offspring from the XY^*DMY*−^ mutated female testcross, we found that it was difficult to obtain a sufficient number of offspring. Therefore, we performed the comparative analysis of the number of matured oocytes between the WT female and XY^*DMY*−^ mutants (N = 10). During 10 days’ embryos collecting, the XY^*DMY*−^ mutated female produced six eggs per day on average; whereas, the WT XX female produced 20 eggs per day (Fig. 5E). The results from F2 phenotypes revealed that the genetic males (XY) of TALENs-induced *DMY* gene disruption mutants all developed into females and laid eggs (DMYΔ11 and DMY+16 mutants). Furthermore, histological analyses demonstrated that all XY mutants developed into females; however, there were significant differences in germ cells and gonad (Fig. 5F). The ovary of XY^*DMY*−^ mutants (12/15) appeared to have fewer oocytes than that of the WT XX female (Fig. 5F). According to the developmental stage of oocytes in medaka, there were equilibrium distributions in each stage of oocytes in the WT XX female ovary, containing cortical alveolar oocytes (CA), vitellogenic oocytes (VO) and mature oocytes (MO). Among 15 XY^*DMY*−^ mutants (15 mutants were identified in the same genotype, fed in the same tank and sampled at the same time), three did not form mature oocytes, with majority of chromatin nucleolar oocytes (CN), several perinucleolar oocytes (PO) and few CA oocytes in the ovary, and no further developmental stage oocytes (Fig. 5F). Twelve of them successfully formed MOs, however, the size of ovary and total number of each developmental stage oocytes were significant smaller and fewer than that of WT XX female (Fig. 5F). This can also explain it is difficult to collect embryos in XY^*DMY*−^ mutants, even there is sometimes no embryos could be collection (Fig. 5E). The analysis of gonadal histology, GSI, and the statistics of mature eggs demonstrated significant differences in the gonadal development and maturity between WT females and XY^*DMY*−^ mutant females.

### Transcriptomic analysis of TALENs-induced *DMY*- mutants and WT

**Figure 6:**
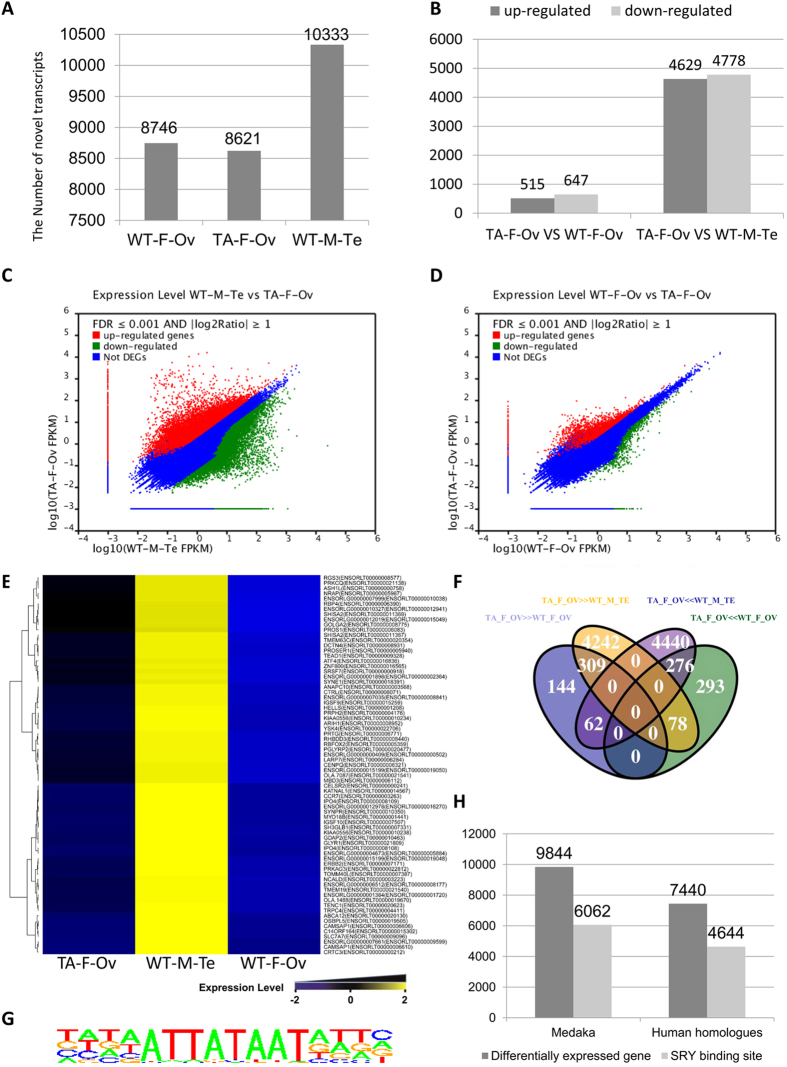
Bioinformatic analyses of RNA-seq data. (**A**) The number of novel transcripts in the RNA-seq data the WT female (WT_F_OV), the wild-type male (WT_M_Te) and XY^*DMY*−^ female medaka (TA_F_OV). (**B**) The differentially expressed transcripts between TA_F_OV and WT_F_OV/WT_M_Te. (**C**) Correlation of gene expression between WT_M_Te and TA_F_Ov. The up- and downregulated genes are shown in red and green, respectively. Non-differentially expressed genes are shown in blue. (**D**) Correlation of gene expression between WT_F_Ov and TA_F_Ov. The up- and downregulated genes are shown in red and green, respectively. Non-differentially expressed genes are shown in blue. (**E**) The cluster of testis-specific expressing transcripts. Cluster analyses of differentially expressed genes among WT_F_Ov, WT_M_Te and TA_F_Ov. The high- and low-expressed genes are shown form yellow to blue, corresponded to the expression level from negative 2 fold to positive 2 fold. (**F**) The differentially expressed transcripts among TA_F_Ov, WT_F_Ov and WT_M_Te. The up- and downregulated genes in WT_M_Te were shown in Fuchsia and yellow, respectively. The up- and downregulated genes in WT_F_Ov are shown in green and purple, respectively. TA_F_Ov ≫ WT_F_Ov means the genes were upregulated in TA_F_Ov. TA_F_Ov ≪ WT_F_Ov means the genes were downregulated in TA_F_Ov. TA_F_Ov ≫ WT_M_Te means genes were upregulated in TA_F_Ov. TA_F_Ov ≪ WT_M_Te means the genes were downregulated in TA_F_Ov. (**G**) SRY binding sites. (**H**) The SRY binding sites analyses of differentially expressed genes among TA_F_Ov, WT_F_Ov and WT_M_Te.

To better explore the transcriptional regulation function of *DMY*, and identify *DMY*-related downstream factors that affect the generation, development and maturation of testis or ovary, we used RNA-seq to analyze the transcriptome of WT_M_TE, WT_F_OV and TA_F_OV after 90 days. For both the WT and *DMY*-mutants, ten samples of gonadal tissues were mixed for library construction. RNA-seq generated 157 million raw reads comprising 10333, 8746 and 8621 transcripts, respectively (Fig. 6A). Using the Ensembl medaka genome database as the reference, 75.8% of the raw reads matched medaka genomic sequences (File S6). Twelve genes were selected randomly from hundreds of different expression transcripts between the gonad of WT and that of *DMY* mutants. The result of real-time quantitative PCR showed that the trends of these genes expression were the same as in the RNA-seq data (File S7). Thus, the RNA-seq information was accurate and reliable.

To isolate *DMY*-related gonad developmental-regulated genes, we conducted a comparative analysis of the three transcriptomic groups. Comparing WT_M_TE with TA_F_OV, there were 9407 differentially expressed transcripts (Data A, Dataset 1). The only difference between WT and TA medaka is the absence of the *DMY* gene, more or less, these differentially expressed genes were attributed to the loss of the *DMY* gene. There were 4629 upregulated genes and 4778 downregulated genes in WT-M-Te (Fig. 6B,C). The functions of these genes were mainly related to cilium morphogenesis (191), cilium organization (152), cilium assembly (152), spermatid differentiation and development, wnt signal pathway (77) (Dataset 1). The master male SD marker, *DMY*^12^,^13^ (*Dmrt1Y,* Uingene20290/Unigene19692), did not expressed in TA_F_OV (Dataset 1), supporting previous observations that *DMY* gene was successfully disrupted in XY^*DMY*−^ mutants (Fig. 4C). The male sex-differentiation or testes maintaining marker *Dmrt1a*^38^ (Unigene42535/Unigene30831), *Gsdf*^39^ (Unigene44032), *Sox9b*^40^ (Unigene18419) were also significantly down-expressed in TA_F_OV (Dataset 1). Interestingly, the majority of genes in wnt signal pathway were differentially expressed between WT_M_TE and TA_F_OV. *Wnt1* (Unigene54610), *Wnt5a* (Unigene50823), *Wnt5b* (Unigene25302), *Wnt9* (Unigene32218), *Wnt11* (CL6243), *PRCK* (CL13707, PREDICTED: *Oryzias latipes* PRKC apoptosis WT1 regulator protein-like), *DKK* (Unigene58048) and *PRKCA* (Unigene17297) were up-expressed in TA_F_OV; *Wnt2* (Unigene 27916), *Wnt8a* (Unigene51222), *FZD6* (CL9539) and *FZD8* (Unigene49832) were down-expressed in TA_F_OV (Dataset 1). These genes also contribute significantly to the molecular supporting the sex-reversal female phenotype of XY^*DMY*−^ mutants (Fig. 5A,B).

Theoretically, a number of differentially expressed genes between the testis (WT_M_TE) and ovary (WT_F_OV) (Data B) might confound Data A. More accurately, Data B must be excluded from Data A. The intersection of Data A and Data B identified the real differentially expressed genes between the WT XY male testis and XY^*DMY*−^ female ovary (Data C, Dataset 2), which were the sum of 309 transcripts, 276 transcripts, 144 transcripts, 293 transcripts, 62 transcripts and 78 transcripts, which are *DMY* -related or affected genes (Fig. 6F). Relative to WT_F_OV, 144 ovary-specific transcripts were downregulated and 293 ovary-specific transcripts were upregulated in male-to-female reversed gonads (Fig. 6F). 62 transcripts in XY^*DMY*−^ female ovary were downregulated relative to WT_M_TE and were upregulated relative to WT_F_OV. 78 transcripts in XY^*DMY*−^ female ovary were upregulated relative to WT_M_TE and were downregulated relative to WT_F_OV. Relative to WT_F_OV and WT_M_TE, 309 transcripts were upregulated in TA_F_OV, and 276 transcripts were downregulated in TA_F_OV (Fig. 6F and Dataset 2). Seventy-three testis-specific transcripts were identified (Fig. 6E and File S8). GO analysis and homologous annotation with human genes is a traditional way to further predict the functions of the genes in Data C (Dataset 2). According to the annotation of Blast2GO, genes of Data C were associated with the regulation of ubiquitination and fertilization. Moreover, a large number of genes, such as ENSORLT00000000529, have not been previously reported.

GO analysis is not a straightforward way to predict the relationships among genes. Sry and *DMY* are transcription factors; theoretically, their downstream genes should have the direct binding regions for Sry or *DMY*. The predicted binding site of human SRY is shown in Fig. 6G. Human homologous genes of data C were scanned for SRY binding sites (Dataset 3). If SRY binding sites could be found in the human homologous genes, we could speculate that their medaka homologous genes may have the *DMY* binding sites or be a directly affected gene. There were 9844 unique genes in medaka that were analyzed: 7440 of which had homologous genes in the human genome. There were 4644 human homologous transcripts that may have more than one potential SRY binding site (Fig. 6H and Dataset 3). A number of potential *DMY* regulated genes, such as SLC25A38, had a potential SRY binding site in the upstream region of its human homolog; and a novel transcript, ENSORLT00000000529, had six potential SRY binding sites in the upstream region of its human homolog. These genes are significant for investigating the transcriptional function of the *DMY* gene.

When we compared TA_F_OV with WT_F_OV, 1163 differentially expressed transcripts (Data D) were found in XY^*DMY*−^ female (Dataset 4). There were 515 upregulated genes and 647 downregulated genes in WT-F-Ov (Fig. 6B,D). Using Blast2GO, the differentially expressed genes were blasted and annotated on biological process (BP), cell components (CC), and molecular function (MF). The genes of Data D are associated with the wnt receptor signaling pathway (Predicted: syntabbulin-like, axin-2-like), ovarian follicle development (Forkhead box O5, Predicted: beta-arrestin-1-like, adenomatous polyposis coli protein-like, ubiquitin-protein ligase E3A-like, bone morphogenetic protein receptor type-1B-like), and the follicle-stimulating hormone signaling pathway (luteinizing hormone receptor, lhr, Predicted: beta-arrestin-1-like) (Dataset 4). In terms of the number and repetitions of the genes, the genes of the wnt signaling pathway were the most significant proportion of Data D. *PRCKA* (CL423) and *DKK* (Unigene31972) were up-expressed in TA_F_OV; *Wnt1* (CL10671), *MAPK8* (CL4166), *FZD6* (CL9539) and *PRCK* (CL13707) were down-expressed in TA_F_OV. These genes, especially *Wnt1* and *PRCK* (CL13707), were down-expressed in XY^*DMY*−^ mutants, compared with WT XX females (Dataset 4). Therefore, we reasonably believed that the wnt signaling pathway is one of the vital pathways on regulating the development and mature of oocytes in XY^*DMY*−^ mutants, why the ovarian function of XY^*DMY*−^ mutants are significant lower than that of WT females. Comprehensive Data A, B, C, and D, greatly promote research into the transcriptional function of the *DMY* gene in medaka, not only in sex reversal, but also in the normal development and maturation of gonads.

## Discussion

### *Nanos3UTR*- TALENs effectively generated a targeted gene mutant line in Medaka

Germline transmission of the knockout genotypes is critical to obtain homozygous gene knockout animals. Generally, a 46.8% mutation rate in the F0 is not ideal for gene disruption. Perhaps this low efficiency should be attributed to the *DMY* gene on the Y chromosome rather than our optimized TALENs. In general, there should be equal numbers of male and female embryos in a generation. This means that the targeting efficiency of *DMY*-TALENs could not beyond be greater than 50%. Indeed, in our results, the efficiency was never more than 50%, even if the concentration of TALENs was increased to 800pg (Fig. 2I). To improve the germline integration efficiency, we incorporated the zebrafish *nanos*-3′UTR into the TALEN construct (Fig. 1A), which was reported to protect mRNA from degradation in primordial germ cells and improve the germ cell targeting efficiency^28^,^41^,^42^. Compared with 9.02% *DMY*-TALEN-induced F1 embryos carrying mutations; there were 37.56% *DMY-*nanos3UTR-TALEN-induced F1 embryos that carried mutations in the *DMY* gene (Fig. 2I and File S2). These results indicated that *DMY-*nanos3UTR-TALEN induced a high proportion, possible a majority, of mutant gametes in the F0 medaka. Thus, the incorporation of the *nanos*-3′UTR into the TALEN construct improved the germ cell targeting efficiency in medaka, permitting the generation of medaka knockout lines.

### A novel program to identify potential off-target sites of TALENs

Specificity is essential to establish precisely targeted gene knockout lines. TALENs have become an accepted tool for targeted mutagenesis, but undesired off-targets, in addition to the targeted genomic region, remain an important issue^15^,^21^,^43^,^44^,^45^,^46^,^47^. Unfortunately, using e-PCR to perform BLAST searches, potential off-target sites were identified in several studies^46^. Using Primer3 and BLAST, potential off-target sites of the *DMY* gene in medaka were identified (File S5). According to this data, BLAST emphasized sequence similarity, but there were few base pair sites of TALENs that could match to potential off-target sites of the genomes. In fact, not only the similarity of sequence, but also that fact that the 0 position of EBEs must be T is very important to TALENs binding site^48^. Using the novel program, 55 candidates’ off-target sites were identified in the medaka genome. We amplified the top predicted potential off-target locus by PCR, sequenced it and found no mutations (File S4). Using our data, our program was more efficient than ePCR to predict TALENs off-target sites. Recently, tools for predicting TALEN off-targets have been developed, such as idTALE (http://idtale.kaust.edu.sa), Paired Target Finder (https://tale-nt.cac.cornell.edu), and TALENoffer^49^. Compared with these, the advantage of our program is its simplicity and reliability. However, a more detailed investigation on the possible off-target effects of TALENs and more accurate program will be needed in the future.

### Mutant phenotypes

Gonadal dysgenesis in 46, XY patients was first noted by Drash *et al.*^4^. Phenotypic female patient with an XY karyotype were initially reported by Kaplan^5^. Several phenotypic female patients with the XY karyotype were evaluated clinically, cytogenetically, hormonally, endoscopically and histologically^6^. The functional study of gonadal dysgenesis in phenotypic female patients with an XY karyotype has been hindered by a lack of animal models with specific mutations, except for the mouse sry mutant^3^,^31^. Using TALENs, two different Y-linked genes were efficiently manipulated in mouse embryonic stem cells (mESCs)^31^ and an *Sry* knockout mouse was generated^3^. The mutant mice are almost completely infertile, although the *Sry* knockout mouse is similar to humans in terms of their physiological phenotype. The phenotype of the *DMY* knockout medaka is also similar to that human XY female syndrome. In particular, a majority of fertile individuals were found in the population. XY^*DMY*−^ female medakas could help in studies of the mechanism of human XY female syndrome in genetics and reproductive biology. Benefiting from the number of embryos or offspring from the parents, fish are good for large sample analysis and for investigating individual differences of human XY female syndrome in populations.

*DMY*, a duplicated copy of *DMRT1*, is identified as the master male SD gene and shows all features of a SD gene in medaka^12^. In this study, we generated both insertions (+16) and deletions (−11) of the TALEN-induced *DMY* mutant line, which developed into females with the XY karyotype (Fig. 5A–C,F). Sex reversal was also reported very early in medaka^50^, which could be induced by steroid hormones or high temperature^51^,^52^. To evaluate whether the sex reversal observed in XY^*DMY*−^ female medaka was caused by *DMY* gene mutation, the best method is rescue of the phenotypes of the XY^*DMY*−^ female medaka. In the former case, the expected mutant *DMY* lacks functionally important motifs of DMY; this mutated Y chromosome could pair with the WT X and Y chromosome to generate females and males, respectively. Crossing with the WT medaka (Fig. 3), produced Y^*DMY*−^Y male mutants in the next generation, which meant that dysfunction of the *DMY* gene was the unique difference between XY^*DMY*−^ female and Y^*DMY*−^Y male. This result confirmed that the sex reversal observed in XY^*DMY*−^ female medakas was caused by a *DMY* gene mutation.

Adults of two phenotypically female mutant lines were evaluated through mutation confirmation, phenotype diagnostic (Fig. 5A) and histological analysis (Fig. 5B,F). Using genomic PCR (Fig. 4B) and FISH of *DMY* gene (Fig. 5C) showing the specific hybridization signal of the Y chromosome, the two phenotypically female mutant lines were identified as genetic males with the XY karyotype. RT-PCR analysis (Fig. 4C) and RNA-seq (Dataset 1) confirmed that XY^*DMY*−^ mutants did not express *DMY* gene, which were different from WT XY individuals. *Gsdf,* co-localized with *DMY* in the same somatic cells in the XY gonads, was expressed exclusively in primordial gonads of only the genetic males^39^. Both the increased number of germ cells (Fig. 4D) and the significantly down-expression of *Gsdf* gene (Unigene44032) implied sex reversal of XY^*DMY*−^ mutants took place in early developmental stages, attributed to the disruption of *DMY* gene. As expected, the TALEN-induced *DMY* knockout medakas had female external and internal specificities. Unlike the *Sry* KO mouse, which did not produce any offspring^3^, the majority of TALEN-induced *DMY* knockout medakas were fertile. Twelve of 15 XY^*DMY*−^ mutants developed functional ovaries; and the ovaries of three females showed incomplete development. There were significant differences among XY^*DMY*−^ mutants, WT female and WT male in GSI (Fig. 5D) in terms of their GSI scores. Histological analysis showed that the ovary of XY^*DMY*−^ mutants displayed a reduced number of oocytes (Fig. 5F). In three of the 15 mutants, no eggs could develop beyond CA oocytes. The other 12 could generate mature eggs, however, the mature oocytes number of XY^*DMY*−^ mutants are significantly fewer than that of WT XX females. Therefore, the ovary function of XY^*DMY*−^ mutants was lower than that of WT females, not only in quality, but also in the quantity (Fig. 5F). To investigate whether there is significant difference in the fertilization of eggs and between XY^*DMY*−^ female and WT female; test crosses were continuously and systemically recorded. During generation of offspring from the XY^*DMY*−^ female testcrosses, we found that it was always difficult to obtain a sufficient number of offspring. After 10 days of continuous monitoring, 6 eggs matured per day from XY^*DMY*−^ mutants; 20 eggs matured per day from WT XX females (Fig. 5E). The results from F2 phenotypes revealed that a reduced number of mature oocytes were one of the most plausible explanations for the reduced fertility in the *DMY* KO medakas. Thus, the XY^*DMY*−^ mutants could develop functional ovaries; however, the development, maturation and fertilization capacity of their eggs were significantly lower than those of the WT female.

### Transcriptomic analysis of TALENs induced *DMY-* mutants and the WT

Natural sex reversal has been reported in medaka^50^, and mutation of the *DMY* gene was identified in several artificial mutants^14^. However, the molecular mechanism of sex reversal, inducing by loss of *DMY*, has not been resolved. RNA-Seq is a recently developed approach for transcriptome profiling that is based on deep-sequencing^32^. RNA-seq could quantify the changing expression levels of each transcript during development and under different conditions. XY^*DMY*−^ mutant female from the F3 generation were used for RNA-seq analysis, which minimized the off-target effects of *DMY*-TALENs. Using the RNA-seq approach, we obtained transcriptome information of XY^*DMY*−^ mutants, and through a comparative analysis of them, revealed the transcriptional function of the *DMY* gene in medaka.

Using RNA-seq, 157 million raw reads were generated from WT_M_TE, WT_F_OV and TA_F_OV libraries. However, only 75.8% of the processed reads were mapped to the reference genome of medaka in the Ensemble database (File S6). This indicated that the medaka genome information requires improvement, and transcriptomic sequencing could revise and promote the improvement of medaka genome. In addition, it implied that it would be better to use independent analysis with no reference genome in the analysis of medaka transcriptome, which we used in this study. The problem with the medaka genomic information had little bearing on the fact that medaka is a good model for human diseases. Although only 7481 of 9844 transcripts could match the medaka genome, 7440 transcripts had homologous genes in the human genome (Fig. 6H). This suggested that medaka might be similar to humans in terms of sex determination and regulation; the medaka transcriptome data could play a role in the analysis of human sex reversal patients.

Medaka sex is primarily determined by the presence or absence of *DMY* gene^12^,^13^. Several studies show that *Dmrt1*^16^,^38^, *Gsdf*^39^ and *Sox9b*^40^ is essential to maintain testis differentiation or regulate testis development. Un-expression of *DMY* (Uingene20290) and lower expression of *Dmrt1a* (Unigene42535), *Gsdf* (Unigene44032) and *Sox9b* (Unigene18419) in RNA-seq analysis provided the molecular basis to the failure of male sex determination, male-to-female sex reversal, and the failure of testis differentiation or development in XY^*DMY*−^ mutants (Dataset 1). Among the differentially expressed genes in TA_F_OV compared with WT_F_OV and TA_F_OV compared with WT_M_TE (Fig. 6F and Dataset 2), we found several potential factors may directly bind SRY and DMY, such as SLC25A38 and ENSORLT00000000529. This validated the transcriptional function of the *DMY* gene. Theoretically, the *DMY* gene, a SD gene, might have a relationship with the sperm production and cilia assembly. However, from our comparative analysis, it was apparent that differences in the expressions of genes involved in these processes represented background differences between the WT ovary and WT testis tissue. This is an essential problem in the transcriptional function of *DMY*, but it beyond the scope of this study. Further, we may investigate developmental stages of gonad or different tissue of the HPG axis to explain the problem.

The main differentially expressed genes between WT_F_OV and TA_F_OV, were genes in the wnt receptor signaling pathway (Predicted: syntabbulin-like, axin-2-like) (Dataset 4). In mammals, beta-arrestin-1-like gene regulated IGF-1 affects human reproductive endocrinology. The medaka homolog of beta-arrestin-1-like gene was detected 10 times in the RNA-seq data. The high expression of the beta-arrestin-1-like gene could provide some clues to the mechanism of the degradation of the testis and the development of the ovary in XY *DMY* mutant females. Lhr and Forkhead box O5 factor were also differentially expressed between TA_F_OV and WT_F_OV. Lhr is the luteinizing hormone receptor, and Forkhead box O5 factor plays an important role in metabolism and cell differentiation. The low expression of lhr and Forkhead box O5 factor might affect the incomplete ovary function of the XY *DMY* mutant female. The majority of genes in wnt signal pathway, such as *Wnt* genes, *PRCK* genes, *FZD* genes and *DKK* were differentially expressed between WT_M_TE and TA_F_OV (Dataset 1), it implied that the wnt signaling pathway is the main regulation pathway during male-to-female sex reversal process. In addition, the genes in wnt signaling pathway, especially *Wnt1* and *PRCK*, were up-expressed in XY^*DMY*−^ mutants compared with WT XY males, and down-expressed compared with WT XX females (Dataset 1 and 4). These results implied that the wnt signaling pathway also contributed primarily to the ovarian development, reduced fertility and ovarian maturation in the XY^*DMY*−^ mutants. Interestingly, *PRCKA* and *DKK* were up-expressed in TA_F_OV, whether compared with WT_M_TE (Dataset 1) or compared with WT_F_OV (Dataset 4). In summary, these results implied that the wnt signaling pathway is the root of sex reversal, the incomplete ovarian function and reduced fertility in the XY^*DMY*−^ mutants.

## Methods

### Ethics Statement on the Use of Animals

The research animals are provided with the best possible care and treatment and are under the care of a specialized technician. All procedures were approved by the Institute of Hydrobiology, Chinese Academy of Sciences, and were conducted in accordance with the Guiding Principles for the Care and Use of Laboratory Animals.

### Medaka husbandry

The Orange strain of medaka was used in this study (Originally from National University of Singapore). Medakas were maintained in aquaria under an artificial 14-h light/10-h dark photoperiod at 26 °C in the Institute of Hydrobiology, Chinese Academy of Science^30^.

### Construction of TALENS

TALEN were assembled and transferred into vectors pCS2-KKR and pCS2-ELD^28^. To improve the germline integration efficiency, the zebrafish *nanos*-3′UTR was separately inserted into the 3′ end of the pCS2-ELD/KKR vector to replace the SV40 UTR using *NotI* and *XbaI*.

### Manipulation of medaka embryos

The final TALEN plasmids were linearized using *Not*1, and the mMessage mMachine SP6 kit (Ambion) was used to synthesize mRNAs. TALENs mRNAs (half left and half right monomer mRNAs) were microinjected into medaka embryos at the one cell stage.

### Detection of mutations in TALEN-targeted medaka embryos

72 hours after microinjection, TALEN targeted embryos were pooled for genomic DNA extraction (10 embryos for each pool). PCR was performed using primers DMY F and DMY R; PCR products were purified from the agarose gel using a gel extraction kit (QIAGEN). Amplicons harboring the targeted gene fragments were sub-cloned into pMD-18T using TA cloning (Takara), and single colonies were examined by PCR using primers DMY F, DMY F1 and DMY R. The PCR conditions were as follows: 5 min at 95 °C; followed by 30 cycles of 15 sec at 95 °C, 20 sec at 52 °C, 30 sec at 72 °C; and a final step of 5 min at 72 °C. PCR products were electrophoresed in a 2% agarose gel and verified by DNA sequencing.

### Off-target analysis

The criteria of the novel program for determining off-target sites were that the 0 position of the EBEs must be T, from 1 to 10 mismatch bases occur in the pairs of EBEs, and the spacer between the two putative EBE regions is less than 100 bp, because it has been suggested that longer spacers interfere with Fok I dimerization.

### Founder Screening

TALEN-injected medaka embryos were raised to sexual maturity. F0 *DMY* mutated females were crossed with wild-type males; and F1 embryos were collected at 72 hours post fertilization. Genomic DNA was extracted from ten randomly pooled embryos to assess mutagenesis at the TALEN-targeted site by PCR and sequencing. F1 embryos were individually collected at 7 days post fertilization (dpf), and genomic DNA was extracted from each individual embryo to assess mutagenesis at the TALEN-targeted site by PCR and sequencing.

### Fluorescence *in situ* Hybridization

FISH was performed using a *DMY* fragment labelled by PCR (Dmy Probe F: 5′-TGCCCAAGTGCTCCC-3′; Dmy Probe R: 5′-CCCCTTTTGTCTGTCCTCT-3′) with digoxigenin-11-dUTP (Boehringer). Standard nick translation using biotin-16-dUTP and digoxigenin-11-dUTP^12^ separately labeled the probe. Before hybridizing with denatured medaka mitotic chromosomes, the probe was denatured and preannealed in the presence of excess genomic DNA. Rhodamine-conjugated avidin and antidigoxigenin (monoclonal)-conjugated fluorescein (Sigma) were used to detect the hybridization sites for the probe simultaneously. 4, 6-diamidino-2-phenylindole (DAPI) was used to counterstain the chromosomes. EASY FISH 1.0 software (Applied Spectral Imaging, Mannheim, Germany) was used to capture and display digitized images of the rhodamine signals on DAPI-stained chromosomes.

### Expression Analysis

The TRIZOL reagent (Invitrogen) was used to extract total RNA from pooled organs of three adult medaka fish, according to the supplier’s recommendation. After DNase treatment, reverse transcription was performed with 2 or 4 mg RNA by using Superscript II reverse transcriptase (Invitrogen) and random primers. cDNA from 10 ng (actin) to 200 ng (adult organs) of total RNA was used for PCR with gene-specific primers: *Ola Actin*, *Ola DMY* (Dmy F1 and PG17.89 primers^12^).

### Histological Analysis

For the histological analysis, identified mutants from the F2 generation of medaka were dissected into head and body segments (N = 15). Each dissected head was used to determine the genetic sex. The body portions were fixed overnight in Bouin’s fixative solution and then embedded in paraffin. Cross-sections were cut serially at 5 μm thickness, and after hematoxylin and eosin (H&E) staining; the images were obtained under a microscope (Eppendorf).

### Library construction and high-throughput sequencing

Medaka gonads (divided into WT-F, WT-M and TA-F, 10 samples pooled in each group, respectively) were collected for RNA extraction. TRIZOL (Invitrogen) performed the total RNA extraction, following the manufacturer’s instructions. RNA library construction was then performed by BGI- Shenzhen, Shenzhen, China then constructed the RNA library. Before library construction, the integrity of RNA samples was confirmed using an Agilent 2100 Bioanalyzer; 10 μg of total RNA was used for isolation of mRNA with Sera-mag Magnetic Oligo (dT) beads from Illumina.

### Quantitative Real-Time PCR

The TRIZOL reagent (Invitrogen) was used to isolate total RNA from embryos. The SYBR Green PCR Master Mix (Applied Biosystems) and the Real-Time PCR System (ABI 7900) measured gene transcription. The *actb1* gene was used as the endogenous control gene for normalizing expression of the target gene. Triplicate technical replicates were performed for duplicate cultures.

### Functional Annotation, Classification, and Enrichment Analysis

Blast2GO software (http://www.blast2go.de) was used to perform BLAST searching, mapping, and annotation of proteins differentially expressed^53^. BINGO 2.44^54^ plug-in in the Cytoscape platform^55^ was used to perform functional enrichment analysis of differentially expressed proteins to determine the significantly enriched GO terms and relevant proteins. Enrichment analysis of GO term assignment was performed in reference to the entire annotated *P. tricornutum* proteome. The corrected P values were derived from a hypergeometric test followed by Benjamini and Hochberg false discovery rate correction. A corrected P < 0.05 was regarded as significant.

## Additional Information

**Accession codes:** All RNA-seq data submited to GEO, GEO accession: GSE62700.

**How to cite this article**: Luo, D. *et al.* Direct production of XY^DMY-^ sex reversal female medaka (*Oryzias latipes*) by embryo microinjection of TALENs. *Sci. Rep.* **5**, 14057; doi: 10.1038/srep14057 (2015).

## Supplementary Material

Supplementary Information

Supplementary Dataset 1

Supplementary Dataset 2

Supplementary Dataset 3

Supplementary Dataset 4

## References

[b1] Sinclair A. H. *et al.* A gene from the human sex-determining region encodes a protein with homology to a conserved DNA-binding motif. Nature 346, 240–244 (1990). 1695712 10.1038/346240a0

[b2] Hawkins J. R. *et al.* Mutational analysis of SRY: nonsense and missense mutations in XY sex reversal. Hum Genet 88, 471–474 (1992). 1339396 10.1007/BF00215684

[b3] Kato T. *et al.* Production of Sry knockout mouse using TALEN via oocyte injection. Sci Rep 3, 3136 (2013). 24190364 10.1038/srep03136 PMC3817445

[b4] Drash A., Sherman F., Hartmann W. H. & Blizzard R. M. A syndrome of pseudohermaphroditism, Wilms’ tumor, hypertension, and degenerative renal disease. J Pediatr 76, 585–593 (1970). 4316066 10.1016/s0022-3476(70)80409-7

[b5] Kaplan E. Gonadal dysgenesis in a phenotypic female with an XY chromosomal constitution. S Afr Med J 53, 552–553 (1978). 675413

[b6] Portuondo J. A. *et al.* Management of phenotypic female patients with an XY karyotype. J Reprod Med 31, 611–615 (1986). 3091820

[b7] Conover D. O. & Kynard B. E. Environmental sex determination: interaction of temperature and genotype in a fish. Science 213, 577–579 (1981). 17794845 10.1126/science.213.4507.577

[b8] Bulmer M. Evolution: sex determination in fish. Nature 326, 440–441 (1987). 3561482 10.1038/326440b0

[b9] Conover D. O. & Heins S. W. Adaptive variation in environmental and genetic sex determination in a fish. Nature 326, 496–498 (1987). 3561487 10.1038/326496a0

[b10] Loukovitis D. *et al.* Quantitative trait loci for body growth and sex determination in the hermaphrodite teleost fish Sparus aurata L. Anim Genet 43, 753–759 (2012). 22497460 10.1111/j.1365-2052.2012.02346.x

[b11] Piferrer F., Ribas L. & Diaz N. Genomic approaches to study genetic and environmental influences on fish sex determination and differentiation. Mar Biotechnol (NY) 14, 591–604 (2012). 22544374 10.1007/s10126-012-9445-4 PMC3419836

[b12] Nanda I. *et al.* A duplicated copy of DMRT1 in the sex-determining region of the Y chromosome of the medaka, Oryzias latipes. Proc Natl Acad Sci USA 99, 11778–11783 (2002). 12193652 10.1073/pnas.182314699 PMC129345

[b13] Matsuda M. *et al.* DMY is a Y-specific DM-domain gene required for male development in the medaka fish. Nature 417, 559–563 (2002). 12037570 10.1038/nature751

[b14] Matsuda M. *et al.* DMY gene induces male development in genetically female (XX) medaka fish. Proc Natl Acad Sci USA 104, 3865–3870 (2007). 17360444 10.1073/pnas.0611707104 PMC1820675

[b15] Otake H. *et al.* The medaka sex-determining gene DMY acquired a novel temporal expression pattern after duplication of DMRT1. Genesis 46, 719–723 (2008). 18821592 10.1002/dvg.20431

[b16] Masuyama H. *et al.* Dmrt1 mutation causes a male-to-female sex reversal after the sex determination by Dmy in the medaka. Chromosome Res 20, 163–176 (2012). 22187367 10.1007/s10577-011-9264-x

[b17] Esvelt K. M. & Wang H. H. Genome-scale engineering for systems and synthetic biology. Mol Syst Biol 9, 641 (2013). 23340847 10.1038/msb.2012.66 PMC3564264

[b18] Bibikova M., Golic M., Golic K. G. & Carroll D. Targeted chromosomal cleavage and mutagenesis in Drosophila using zinc-finger nucleases. Genetics 161, 1169–1175 (2002). 12136019 10.1093/genetics/161.3.1169 PMC1462166

[b19] Kim Y. G., Cha J. & Chandrasegaran S. Hybrid restriction enzymes: zinc finger fusions to Fok I cleavage domain. Proc Natl Acad Sci USA 93, 1156–1160 (1996). 8577732 10.1073/pnas.93.3.1156 PMC40048

[b20] Kim S. *et al.* Preassembled zinc-finger arrays for rapid construction of ZFNs. Nat Methods 8, 7 (2011). 21191366 10.1038/nmeth0111-7a

[b21] Hockemeyer D. *et al.* Genetic engineering of human pluripotent cells using TALE nucleases. Nat Biotechnol 29, 731–734 (2011). 21738127 10.1038/nbt.1927 PMC3152587

[b22] Huang P. *et al.* Heritable gene targeting in zebrafish using customized TALENs. Nat Biotechnol 29, 699–700 (2011). 21822242 10.1038/nbt.1939

[b23] Li T. *et al.* TAL nucleases (TALNs): hybrid proteins composed of TAL effectors and FokI DNA-cleavage domain. Nucleic Acids Res 39, 359–372 (2011). 20699274 10.1093/nar/gkq704 PMC3017587

[b24] Christian M. *et al.* Targeting DNA double-strand breaks with TAL effector nucleases. Genetics 186, 757–761 (2010). 20660643 10.1534/genetics.110.120717 PMC2942870

[b25] Ishino S. *et al.* Nucleotide sequence of the meso-diaminopimelate D-dehydrogenase gene from Corynebacterium glutamicum. Nucleic Acids Res 15, 3917 (1987). 3588313 10.1093/nar/15.9.3917 PMC340791

[b26] Hwang W. Y. *et al.* Efficient genome editing in zebrafish using a CRISPR-Cas system. Nat Biotechnol 31, 227–229 (2013). 23360964 10.1038/nbt.2501 PMC3686313

[b27] Cong L. *et al.* Multiplex genome engineering using CRISPR/Cas systems. Science 339, 819–823 (2013). 23287718 10.1126/science.1231143 PMC3795411

[b28] Liu Y. *et al.* Inheritable and Precise Large Genomic Deletions of Non-Coding RNA Genes in Zebrafish Using TALENs. PLoS One 8, e76387 (2013). 24130773 10.1371/journal.pone.0076387 PMC3794983

[b29] Liu Y. *et al.* A highly effective TALEN-mediated approach for targeted gene disruption in Xenopus tropicalis and zebrafish. Methods 69, 58–66 (2014). 24556556 10.1016/j.ymeth.2014.02.011

[b30] Qiu C. *et al.* Efficient knockout of transplanted green fluorescent protein gene in medaka using TALENs. Mar Biotechnol (NY) 16, 674–683 (2014). 25056495 10.1007/s10126-014-9584-x

[b31] Wang H. *et al.* TALEN-mediated editing of the mouse Y chromosome. Nat Biotechnol 31, 530–532 (2013). 23666012 10.1038/nbt.2595 PMC3681814

[b32] Wang Z., Gerstein M. & Snyder M. RNA-Seq: a revolutionary tool for transcriptomics. Nat Rev Genet 10, 57–63 (2009). 19015660 10.1038/nrg2484 PMC2949280

[b33] Ansai S. *et al.* Efficient targeted mutagenesis in medaka using custom-designed transcription activator-like effector nucleases. Genetics 193, 739–749 (2013). 23288935 10.1534/genetics.112.147645 PMC3583995

[b34] Miller J. C. *et al.* A TALE nuclease architecture for efficient genome editing. Nat Biotechnol 29, 143–148 (2011). 21179091 10.1038/nbt.1755

[b35] Kobayashi T. *et al.* Two DM domain genes, DMY and DMRT1, involved in testicular differentiation and development in the medaka, Oryzias latipes. Dev Dyn 231, 518–526 (2004). 15376325 10.1002/dvdy.20158

[b36] Otake H. *et al.* Wild-derived XY sex-reversal mutants in the Medaka, Oryzias latipes. Genetics 173, 2083–2090 (2006). 16702419 10.1534/genetics.106.058941 PMC1569717

[b37] Kagawa H., Young G. & Nagahama Y. Relationship between seasonal plasma estradiol-17 beta and testosterone levels and *in vitro* production by ovarian follicles of amago salmon (Oncorhynchus rhodurus). Biol Reprod 29, 301–309 (1983). 6640021 10.1095/biolreprod29.2.301

[b38] Hornung U., Herpin A. & Schartl M. Expression of the male determining gene dmrt1bY and its autosomal coorthologue dmrt1a in medaka. Sex Dev 1, 197–206 (2007). 18391530 10.1159/000102108

[b39] Shibata Y. *et al.* Expression of gonadal soma derived factor (GSDF) is spatially and temporally correlated with early testicular differentiation in medaka. Gene Expr Patterns 10, 283–289 (2010). 20601164 10.1016/j.gep.2010.06.005

[b40] Nakamura S. *et al.* Sox9b/sox9a2-EGFP transgenic medaka reveals the morphological reorganization of the gonads and a common precursor of both the female and male supporting cells. Mol Reprod Dev 75, 472–476 (2008). 17474097 10.1002/mrd.20764

[b41] Koprunner M., Thisse C., Thisse B. & Raz E. A zebrafish nanos-related gene is essential for the development of primordial germ cells. Genes Dev 15, 2877–2885 (2001). 11691838 10.1101/gad.212401 PMC312811

[b42] Mishima Y. *et al.* Differential regulation of germline mRNAs in soma and germ cells by zebrafish miR-430. Curr Biol 16, 2135–2142 (2006). 17084698 10.1016/j.cub.2006.08.086 PMC1764209

[b43] Mussolino C. & Cathomen T. On target? Tracing zinc-finger-nuclease specificity. Nat Methods 8, 725–726 (2011). 21878917 10.1038/nmeth.1680

[b44] Mussolino C. *et al.* A novel TALE nuclease scaffold enables high genome editing activity in combination with low toxicity. Nucleic Acids Res 39, 9283–9293 (2011). 21813459 10.1093/nar/gkr597 PMC3241638

[b45] Tesson L. *et al.* Knockout rats generated by embryo microinjection of TALENs. Nat Biotechnol 29, 695–696 (2011). 21822240 10.1038/nbt.1940

[b46] Lei Y. *et al.* Efficient targeted gene disruption in Xenopus embryos using engineered transcription activator-like effector nucleases (TALENs). Proc Natl Acad Sci USA 109, 17484–17489 (2012). 23045671 10.1073/pnas.1215421109 PMC3491516

[b47] Osborn M. J. *et al.* TALEN-based gene correction for epidermolysis bullosa. Mol Ther 21, 1151–1159 (2013). 23546300 10.1038/mt.2013.56 PMC3677309

[b48] Boch J. *et al.* Breaking the code of DNA binding specificity of TAL-type III effectors. Science 326, 1509–1512 (2009). 19933107 10.1126/science.1178811

[b49] Grau J., Boch J. & Posch S. TALENoffer: genome-wide TALEN off-target prediction. Bioinformatics 29, 2931–2932 (2013). 23995255 10.1093/bioinformatics/btt501

[b50] Yamamoto T. Artificial induction of functional sex-reversal in genotypic females of the medaka (Oryzias latipes). J Exp Zool 137, 227–263 (1958). 13575738 10.1002/jez.1401370203

[b51] Paul-Prasanth B., Shibata Y., Horiguchi R. & Nagahama Y. Exposure to diethylstilbestrol during embryonic and larval stages of medaka fish (Oryzias latipes) leads to sex reversal in genetic males and reduced gonad weight in genetic females. Endocrinology 152, 707–717 (2011). 21239430 10.1210/en.2010-0812

[b52] Hattori R. S. *et al.* Temperature-dependent sex determination in Hd-rR medaka Oryzias latipes: gender sensitivity, thermal threshold, critical period, and DMRT1 expression profile. Sex Dev 1, 138–146 (2007). 18391524 10.1159/000100035

[b53] Conesa A. *et al.* Blast2GO: a universal tool for annotation, visualization and analysis in functional genomics research. Bioinformatics 21, 3674–3676 (2005). 16081474 10.1093/bioinformatics/bti610

[b54] Maere S., Heymans K. & Kuiper M. BiNGO: a Cytoscape plugin to assess overrepresentation of gene ontology categories in biological networks. Bioinformatics 21, 3448–3449 (2005). 15972284 10.1093/bioinformatics/bti551

[b55] Shannon P. *et al.* Cytoscape: a software environment for integrated models of biomolecular interaction networks. Genome Res 13, 2498–2504 (2003). 14597658 10.1101/gr.1239303 PMC403769

