# Supplementary material for: Direct production of XYDMY− sex reversal female medaka (Oryzias latipes) by embryo microinjection of TALENs
Source: Sci Rep. 2015 Sep 14;5:14057. doi: 10.1038/srep14057 (PMC4568496; doi:10.1038/srep14057)
Supplement: Supplementary Information [file srep14057-s1.pdf]

Direct production of XY $^{DMY-}$ sex reversal female medaka (*Oryzias latipes*) by embryo microinjection of TALENs

Daji Luo[1, 2], Yun Liu[3], Ji Chen [2], Xiaoqin Xia [2], Mengxi Cao [2], Bin Cheng [1], Xuejuan Wang [1], Wuming Gong [4], Chao Qiu [1], Yunsheng Zhang [2], Christopher Hon Ki Cheng [3], Zuoyan Zhu [2] and Wei Hu [2, *]

[1] Department of Genetics, School of Basic Medical Sciences, Wuhan University, Wuhan, Hubei, P. R. China, 430071;

[2] State Key Laboratory of Freshwater Ecology and Biotechnology, Institutes of Hydrobiology, Chinese Academy of Sciences, Wuhan, Hubei, P. R. China, 430072;

[3] School of Biomedical Sciences, The Chinese University of Hong Kong, Shatin, Hong Kong, P. R. China.

[4] Lillehei Heart Institute, University of Minnesota, Minneapolis, USA

[*] Correspondence and requests for materials should be addressed to Wei Hu (email: huwei@ihb.ac.cn)

File S1- Analysis of *DMY*-TALENs microinjected embryos

A. Developmental analysis of *DMY*-TALENs microinjected embryos

Concentration		Normal	Abnormal	Dead	Total embryos
**200pg**	**Exp1**	35	0	1	35
	**Exp2**	32	0	0	32
	**Exp3**	28	0	0	28
**400pg**	**Exp1**	57	2	3	62
	**Exp2**	54	2	2	58
	**Exp3**	48	1	4	53
**600pg**	**Exp1**	88	3	7	98
	**Exp2**	79	1	6	86
	**Exp3**	61	5	15	81
**800pg**	**Exp1**	18	6	28	52
	**Exp2**	22	12	22	56
	**Exp3**	25	10	27	62

B. Mutation rate of *DMY*-TALENs microinjected embryos

Concentration	Exp1	Exp2	Exp3	Average mutation rate	SD
**200pg**	6.25%	6.45%	3.23%	5.31%	0.01807
**400pg**	25.00%	25.81%	21.88%	24.23%	0.020765
**600pg**	46.88%	38.71%	40.63%	42.07%	0.042701
**800pg**	46.88%	46.88%	45.16%	46.30%	0.009894

File S2- Analysis of *DMY*-UTR-TALENs microinjected embryos

A. Developmental analysis of *DMY*-UTR-TALENs microinjected embryos

Concentration		Normal	Abnormal	Dead	Total embryos
**200pg**	**Exp1**	40	0	0	40
	**Exp2**	35	0	0	35
	**Exp3**	37	0	0	37
**400pg**	**Exp1**	39	1	1	41
	**Exp2**	35	1	2	38
	**Exp3**	39	2	2	43
**600pg**	**Exp1**	35	1	3	39
	**Exp2**	38	2	2	42
	**Exp3**	36	2	2	40
**800pg**	**Exp1**	22	6	20	48
	**Exp2**	20	8	18	46
	**Exp3**	21	9	19	49

B. Mutation rate of *DMY*-UTR-TALENs microinjected embryos

Concentration	Exp1	Exp2	Exp3	Average mutation rate	SD
**200pg**	6.45%	6.67%	6.45%	6.52%	0.001242
**400pg**	25.00%	29.03%	23.33%	25.79%	0.029301
**600pg**	40.63%	41.94%	43.33%	41.96%	0.013544
**800pg**	50.00%	48.39%	46.88%	48.42%	0.015628

File S3. Mismatch seq of *DMY* gene in genome

Subject	Span	Start1	End1	Mis1	Match_Seq1	Start2	End2	Mis2	Match_Seq2	Sum_MM
scaffold1535	17	25405	25422	0	TAGTCCAGAGGCCTCGTC	25439	25456	0	GTGAAGAATGAAGCCGGA	0
ultracontig107	17	171535	171552	0	TAGTCCAGAGGCCTCGTC	171569	171586	1	GTGAAGAATGAAGCTGGA	1
3	12	36197357	36197374	3	TCGTCCAGAGTCTTCGTC	36197386	36197403	4	GTGACGAAGGAAGTCAGA	7
15	1	9611512	9611529	5	TTTTCCAGATGGCTTGTC	9611530	9611547	4	GTGATGAATGAGGCGGCA	9
15	60	14478145	14478162	4	TAGTCCATAGGCTCTGTC	14478222	14478239	5	CTGTTGAAGGAAGCTGGA	9
15	88	22629616	22629633	4	TAGTTCAGAGGACTGGTG	22629721	22629738	5	CAGAAGATTGAAGCGGCA	9
16	49	302443	302460	5	TAGTTCTGTGGCCTCACC	302509	302526	4	CTTAAGAATGAAACCAGA	9
17	54	30925606	30925589	5	TGGTCCTCGGTCCTCGTC	30925535	30925518	4	GTGTAGAAAGAAGACTGA	9
20	77	4926137	4926154	5	TAGTCCAAAAACCTGGTA	4926231	4926248	4	CTGAAGCAGGAAGCCTGA	9
4	77	26072027	26072044	5	TAGGTCAGAGAGCTCTTC	26072121	26072138	4	GTTAATAATAAAGCCGAA	9
4	85	3503679	3503696	5	TGGCCCAGAGGCTCCATC	3503781	3503798	4	CTGTAGAAGGAAGCTGGA	9
5	58	19144433	19144416	4	TAGTCCAGATGCTCCGTA	19144358	19144341	5	CTGAAAGATGAAGCTGCA	9
9	38	4858611	4858594	4	TAGTCAAGAGGGGTCGGC	4858556	4858539	5	GTCAAGAGTGCAGCAGCA	9
10	100	18240628	18240645	5	TAGTTGGGAGTCCTCCTC	18240745	18240762	5	GTGAAAAATGTGTCTGGA	10
10	93	11445563	11445580	5	TAGTAGGGACTCCTCGTC	11445673	11445690	5	GAGATGAAGGAGGCAGGA	10
12	65	19216392	19216409	5	TAGTCTAAAGACGTCCTC	19216474	19216491	5	GAGAAGAAGGAAGACCCA	10
13	51	24810791	24810808	5	TCCTCCAGCGGCCTTGTT	24810859	24810876	5	GAAAACAATGAAGTTGGA	10
14	93	9245601	9245618	5	TGGTCGATAAGCCTCATC	9245711	9245728	5	ATGAAGAGCGAAGGCCGA	10
15	24	15029228	15029245	5	TATTCCAGAGGCTTGGGG	15029269	15029286	5	GTCAAGACAGAAGGAGGA	10
15	26	6978961	6978944	5	TAGTCCAGAGTCTTCACT	6978918	6978901	5	CTGAAGATGGAAGCAGAA	10
15	40	1340005	1340022	5	TGGACCAGAGGCCTGAGC	1340062	1340079	5	TTGAGGATAGCAGCCGGA	10
16	13	21231925	21231908	5	TAATCCAAACGCTTCTTC	21231895	21231878	5	TTGCAGAATGAAGTAGAA	10
16	15	13648743	13648760	5	TTGTCCAGGAACCTCATC	13648775	13648792	5	GCTGAGAATGCAGCCAGA	10
16	73	19887374	19887391	5	TAGGTCAGAGTCCTCGCT	19887464	19887481	5	GTTGAGAATTAAGGAGGA	10
16	94	17991283	17991300	5	TTCTTCAGAGACCTCATC	17991394	17991411	5	GTCAACAATAAAGCCACA	10
17	38	30706131	30706114	5	TTGGCCAGAGGACGCGGC	30706076	30706059	5	GTGGAGACAGAAACTGGA	10
20	6	12281682	12281665	5	TATTCCAGGGGCGACGAC	12281659	12281642	5	CAGTAGAATGAAGCCTCA	10
21	44	16629309	16629292	5	TCCTCCGCAGGCCTCGAC	16629248	16629231	5	GTGAAGGCGGAAGCGGAA	10
23	41	1158397	1158414	5	TCCATCAGAGGCCACGTC	1158455	1158472	5	GTTAAAACTGAGGCAGGA	10

3	54	31811293	31811310	5	TATCCAAGAGGCTTTGTC	31811364	31811381	5	GTCAAACCTGAAGCAGGA	10
3	64	7659857	7659874	5	TTGTGCAGCTGCCTCTTC	7659938	7659955	5	GTGAAGAAGAAAGAAAGA	10
4	30	18941030	18941047	5	TAGACCTGACAGCTCGTC	18941077	18941094	5	GGAAAAAATGAAATCGGA	10
4	63	9288293	9288310	5	TAGCCCTGATTCCTCGCC	9288373	9288390	5	GTGAAAACAGAAGCCCAA	10
4	86	21922241	21922224	5	TAGCTCTGCAGCCTCGTC	21922138	21922121	5	GTAAACAATGGAGCTGCA	10
5	45	33730066	33730083	5	TTCCCCAGAAGCGTCGTC	33730128	33730145	5	CTGAGGAAGGAAGCAGAA	10
5	79	9163864	9163881	5	TGGTCCAGAGCCCTCTGA	9163960	9163977	5	GTGAACAAAGAAGGAGCA	10
5	97	8639758	8639775	5	TAGACAAGACACCTCTTC	8639872	8639889	5	GGGAAGACTGAATCTGCA	10
6	2	17717317	17717334	5	TAGCCCATCGGGCTCGGC	17717336	17717353	5	CAGAAGAAAGAAGCTGCA	10
6	34	7035613	7035630	5	TTGTCCTTAGGCCTCTTT	7035664	7035681	5	GTTCAGAATGCACCCTGA	10
6	56	12066847	12066830	5	TAGCTCAAAGGCCTTGTG	12066774	12066757	5	GTGATGAAGGATCCAGGA	10
6	95	13846644	13846661	5	TGATTCAGAGGCATCGTG	13846756	13846773	5	GCTGAGAATGAAGTGGGA	10
7	66	11600912	11600929	5	TAATCCAGAGGCCATCTG	11600995	11601012	5	GGAAAGAAAGAAGCCAAA	10
8	41	23642320	23642337	5	TAGTCCAGAGGCAATGCA	23642378	23642395	5	TTGCAGAATGGAGCAAGA	10
8	47	7846284	7846301	5	TATCCAAGAGGCTTTGTC	7846348	7846365	5	GGGAGGAGTCAGGCCGGA	10
8	52	16640624	16640607	5	TCCTCCAGATGCTTCATC	16640555	16640538	5	GTGACGAAATAAAACGGA	10
8	81	23051150	23051133	5	TATACCAGCGACCGCGTC	23051052	23051035	5	GAGACGAATGAAGGCAAA	10
9	5	22638091	22638108	5	TGATCCAGAGGCCAGCTC	22638113	22638130	5	GAGGAAAATGAAGACGTA	10
9	94	18472017	18472034	5	TTGTGAAGTGGCCTTGTC	18472128	18472145	5	CTTAAAAATGACGCAGGA	10
scaffold2835	32	8513	8530	5	TACTTTAGACGCCGCGTC	8562	8579	5	GCGAGTCCTGAAGCCGGA	10
scaffold5477	66	2839	2822	5	TAATGCAGCCTCCTCGTC	2756	2739	5	ATGAAGGTTGAAGCCAAA	10
scaffold907	67	61912	61929	5	TAATGCAGCCTCCTCGTC	61996	62013	5	ATGAAGGTTGAAGCCAAA	10
scaffold907	67	63384	63401	5	TAATGCAGCCTCCTCGTC	63468	63485	5	ATGAAGGTTGAAGCCAAA	10
ultracontig101	11	52819	52802	5	TAGTCCATGGGCGGCGCC	52791	52774	5	GTGAAGAAAAAGGATGGA	10
ultracontig115	3	4532294	4532277	5	TACTCCTGAAGCCTTTTC	4532274	4532257	5	TGGAAGAATATAGCCGCA	10
ultracontig257	24	630660	630643	5	TAATCCTGAGCCCTCCTG	630619	630602	5	GAGAAGAAATAAGCTGCA	10
scaffold907	67	61912	61929	5	TAATGCAGCCTCCTCGTC	61996	62013	5	ATGAAGGTTGAAGCCAAA	10

File S4-Mismatch analysis of *DMY*-TALEN sites

Subject 1: Dmrt1 , ultracontig107

Primer:

Mis-107F 5'-CACTGTCCTTGAAGGTGTTTCTC-3'

Mis-107R 5'-TCCGATCAGAATGAACCCCAGTG-3'

Prediction target 1:

TAGTCCAGAGGCCTCGTCCGGCCCTGAAGTGACGGTGAAGAATGAAGCTGGA

Sequencing data:

CACTGTCCTTGAAGGTGTTTCTCTATATCTGTCAAAATCAAACCATCATCAATCATCAAA
CCAAACCTCATTTGTACAGATGTGTCTCTGCTCAGGTCGCCTTGAGGAGGCAGCAGGCT
CAAGAGGAGGAGCTTGGGATTTGTAGTCCAGAGGCCTCGTCCGGCCCTGAAGTGACGGT
GAAGAATGAAGCTGGAGCCGACTGTCTCTTCTCCATGGAGGGGCGATCCGGCACGCCAG
GCGTCCCCCCCAACCCTCTGTCTGCCGCAGGTACAGCTTTTCACTGGGGTTCATTCTGAT
CGGACTTTTCCCAAA

Subject 2: Chromosome: MEDAKA1:3:36196800:36198000:1

Primer:

Mis-Chr3F 5'-AATCAGCTCGCTGCTCCTGGTTC-3'

Mis-Chr3R 5'-CCACTCCCACAGCCACTTAAATC-3'

Prediction mismatch target 2:

TCGTCCAGAGTCTTCGTCAGCGTTTGACGGTGACGAAGGAAGTCAGA

Sequencing data:

TAATCAGCTCGCTGCTCCTGGTTCACATGAAACGCAAAGACGATAAACATAAGAGTTCT
GATGTGTCAAATGGTTTTTATTGGATTATTTTGTGAAACTACAATAAAATCATCAAAGGT
TCTGAAGGGAACCGGCATTTTTAGAGCATCACTTCTCCTTTTATTTCAAATGTTTCCAAA
TAAAAGCAGATCTCATGTCCCCAAAACGCCCGTGTTTAGGTTTCTCAGCCTCTGACCACA
GTAGGAACATTCAATACCACCTTAACTACAGCTACTAGCTGCAGAAACCCTGTTAACCA
ACCCAGATGATCTGGGAGTTTCTTGCTGCTTTCCTCCGTCATTAATTGTTGTTTTTCCCTT
AAAAAAGATTTTATGAAGTGAGCATCTTCGTCCAGAGTCTTCGTCAGCGTTTGACGGTG
ACGAAGGAAGTCAGAGGAGCTTGCATGAAGTCAGAGCTGCCGAGCTCCACAGGCACCT
GAAGGCAACACGAGTTCATGATGGATTTAAGTGGCTGTGGGAGTGGTTCCCAAA

File S5-Mismatch prediction of DMY TALEN L and R sites using BLAST

A. Mismatch prediction of DMY TALEN L sites using BLAST

blastn

RID: 1FFC9U95015

Database: GPIPE/8090/100/ref_top_level

Fields: query id, subject ids, % identity, alignment length, mismatches, gap opens, q. start, q. end, s. start, s. end, evalue, bit score

1526 hits found

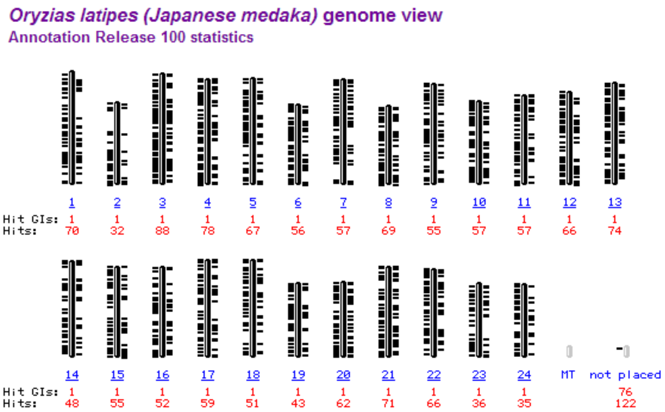

B. Mismatch prediction of DMY TALEN R sites using BLAST

blastn

RID: 1FGEH5WM014

Database: GPIPE/8090/100/ref_top_level

Fields: query id, subject ids, % identity, alignment length, mismatches, gap opens, q. start, q. end, s. start, s. end, evalue, bit score

3493 hits found

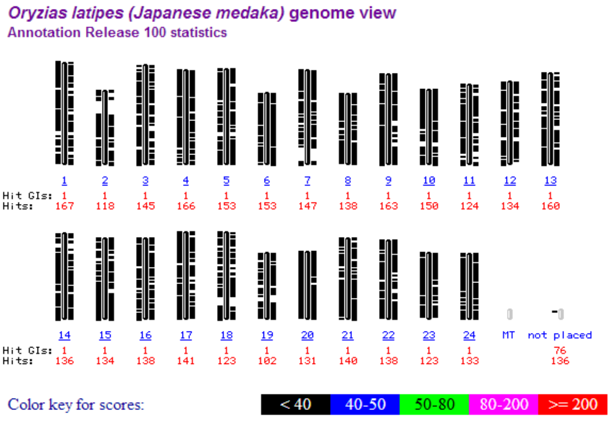

File S6- The raw reads of RNA seq. matched species genomic sequences

Species	Gene Numbers	Percentage
**Oryzias latipes**	58114	75.93%
**Oreochromis niloticus**	8145	10.64%
**Takifugu rubripes**	2098	2.74%
**Danio rerio**	1496	1.95%
**Tetraodon nigroviridis**	1460	1.91%
**other**	5228	6.83%

From BGI Transcriptome Assembly Analysis Report

File S7- Quantitative analyses of gene expression levels in wild-type and *DMY* mutants

A. Genes and primers list in quantitative analyses

Detector	Primer Name	Primer sequence (5'-3')	Amplicon size(bp)
**VASN**	VASN-F	TGTCATCATTTCACGGCAGGT	135
	VASN-R	ACAATAGGGCGGCGGTCAG	
**TEKT2**	TEKT2-F	ATTGATAAATCCTGCCTGACTC	139
	TEKT2-R	CCTCGGCTTGGACCACAT	
**BAZ1B**	BAZ1B-F	CGCAGTGGACGAAGAGTGT	147
	BAZ1B-R	TTGTCGCTGGACGGTGAGT	
**EAF1**	EAF1-F	GCGAATTTGTACTGGAGAAGC	109
	EAF1-R	GCTGGAACGAACCGACTGC	
**SRSF7**	SRSF7-F	TCTCTGTGGGTCCCGTGTT	138
	SRSF7-R	GCAGTCGTAGGCATAGTGGC	
**CCR7**	CCR7-F	TCTGGGTGATGGCACTGAT	126
	CCR7-R	CTATTTGGCTGGCTTGGAT	
**GAS1B**	GAS1B-F	CTCCGAATCCCAACCACC	133
	GAS1B-R	CACTTCTTCCGCTCACCACT	
**CYP21A2**	CYP21A2-F	TCCAAAGTACAGCGACAGAC	144
	CYP21A2-R	CTTGGGAATGAAGAAACCTG	
**SLC25A38B**	SLC25A38B-F	AAGAACAACGCCAAACCG	122
	SLC25A38B-R	ATGCAGCGCACGAACGAC	
**RAPGEF6**	RAPGEF6-F	TCCAACCCTGCTGACATTC	112
	RAPGEF6-R	CGTGGGCAACAACATCCTT	
**ZBTB16B**	ZBTB16B-F	CCAACTCAACAATCCCACTCA	109
	ZBTB16B-R	GAACTCCTGGCCGTCCACT	
**EGFR**	EGFR-F	GTTCAAGAAGGTCAGGGTGC	137
	EGFR-R	GATTTGCTTTGGGTGATGTG	

B. Quantitative analyses of gene expression levels in wild-type and *DMY* mutants

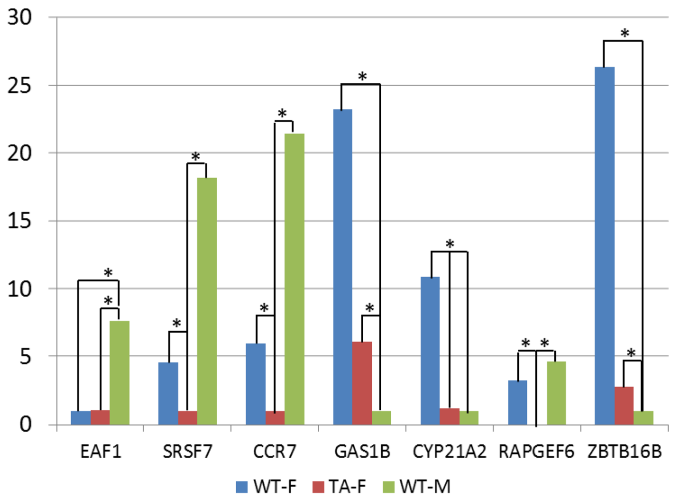

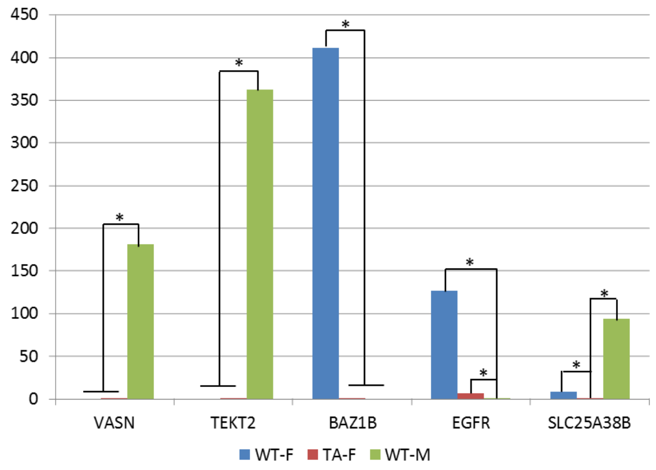

File S8. Seventy-three testis-specific transcripts

Symbol (Accession No.)
CAMSAP1(ENSORLT00000006610)
ENSORLG00000007661(ENSORLT00000009599)
SLC7A7(ENSORLT00000009096)
C14ORF164(ENSORLT00000015302)
CAMSAP1(ENSORLT00000006606)
OSBPL5(ENSORLT00000019505)
ABCA12(ENSORLT00000020130)
TRPC4(ENSORLT00000004411)
TENC1(ENSORLT00000020623)
OLA.1488(ENSORLT00000019670)
ENSORLG00000001384(ENSORLT00000001720)
TMEM19(ENSORLT00000021540)
ENSORLG00000006512(ENSORLT00000008177)
NCALD(ENSORLT00000003223)
TOMM40L(ENSORLT00000007387)
PRKAG3(ENSORLT00000022812)
ERBB2(ENSORLT00000007171)
ENSORLG00000015199(ENSORLT00000019048)
ENSORLG00000004673(ENSORLT00000005884)
IPO4(ENSORLT00000008108)
GLYR1(ENSORLT00000021809)
GDAP2(ENSORLT00000010463)
KIAA0556(ENSORLT00000010238)
SH3GLB1(ENSORLT00000007331)
IGSF10(ENSORLT00000007507)
MYO18B(ENSORLT00000001441)
SYNPR(ENSORLT00000010350)
ENSORLG00000012978(ENSORLT00000016270)
IPO4(ENSORLT00000008109)
CCR7(ENSORLT00000003263)
KATNAL1(ENSORLT00000014567)
CELSR2(ENSORLT00000000241)
MBD3(ENSORLT00000006112)
OLA.7087(ENSORLT00000021541)
ENSORLG00000015199(ENSORLT00000019050)
CENPQ(ENSORLT00000006321)
LARP7(ENSORLT00000006284)
ENSORLG00000000409(ENSORLT00000000502)
PGLYRP2(ENSORLT00000020477)
RBFOX2(ENSORLT00000005359)
RHBDD3(ENSORLT00000008440)
PRTG(ENSORLT00000006771)

YSK4(ENSORLT00000022706)
ARIH1(ENSORLT00000008952)
KIAA0556(ENSORLT00000010234)
PRPH2(ENSORLT00000004176)
HELLS(ENSORLT00000001208)
IGSF9(ENSORLT00000015259)
ENSORLG00000007035(ENSORLT00000008841)
CTRL(ENSORLT00000008071)
ANAPC10(ENSORLT00000003568)
SYNE1(ENSORLT00000018391)
ENSORLG00000001898(ENSORLT00000002364)
SRSF7(ENSORLT00000000918)
ZNF800(ENSORLT00000016565)
ATF4(ENSORLT00000016836)
TEAD1(ENSORLT00000009328)
PROSER1(ENSORLT00000005940)
DCTN4(ENSORLT00000008931)
TMEM63C(ENSORLT00000020354)
SHISA2(ENSORLT00000011367)
PROS1(ENSORLT00000006083)
GOLGA2(ENSORLT00000008775)
ENSORLG00000012019(ENSORLT00000015049)
SHISA2(ENSORLT00000011369)
ENSORLG00000010327(ENSORLT00000012941)
RBP4(ENSORLT00000006390)
ENSORLG00000007999(ENSORLT00000010038)
NRAP(ENSORLT00000005987)
ASH1L(ENSORLT00000000758)
PRKCQ(ENSORLT00000021138)
RGS3(ENSORLT00000008577)
CRTC3(ENSORLT00000000212)
